# Specification of Neuronal Identities by Feedforward Combinatorial Coding 

**DOI:** 10.1371/journal.pbio.0050037

**Published:** 2007-02-06

**Authors:** Magnus Baumgardt, Irene Miguel-Aliaga, Daniel Karlsson, Helen Ekman, Stefan Thor

**Affiliations:** Division of Molecular Genetics, Department of Physics, Chemistry and Biology, Linkoping University, Linkoping, Sweden; Cambridge University, United Kingdom

## Abstract

Neuronal specification is often seen as a multistep process: earlier regulators confer broad neuronal identity and are followed by combinatorial codes specifying neuronal properties unique to specific subtypes. However, it is still unclear whether early regulators are re-deployed in subtype-specific combinatorial codes, and whether early patterning events act to restrict the developmental potential of postmitotic cells. Here, we use the differential peptidergic fate of two lineage-related peptidergic neurons in the *Drosophila* ventral nerve cord to show how, in a feedforward mechanism, earlier determinants become critical players in later combinatorial codes. Amongst the progeny of neuroblast 5–6 are two peptidergic neurons: one expresses FMRFamide and the other one expresses Nplp1 and the dopamine receptor DopR. We show the HLH gene *collier* functions at three different levels to progressively restrict neuronal identity in the 5–6 lineage. At the final step, *collier* is the critical combinatorial factor that differentiates two partially overlapping combinatorial codes that define FMRFamide versus Nplp1/DopR identity. Misexpression experiments reveal that both codes can activate neuropeptide gene expression in vast numbers of neurons. Despite their partially overlapping composition, we find that the codes are remarkably specific, with each code activating only the proper neuropeptide gene. These results indicate that a limited number of regulators may constitute a potent combinatorial code that dictates unique neuronal cell fate, and that such codes show a surprising disregard for many global instructive cues.

## Abstract 2

By studying the differential peptidergic fate of two lineage-related neurons in the*Drosophila* ventral nerve cord, the authors provide deeper insights into how, in a feedforward mechanism, earlier developmental determinants become critical players in later combinatorial codes defining cell identity.

## Author Summary

The nervous system contains a daunting number of different cell types, perhaps as many as 10,000 in mammals, far outnumbering regulatory genes in many animal species. Studies of the determinants of cell fate in many systems during the last decade have supported the conclusion that cell fate is not determined by any one regulatory gene, but results from the combinatorial action of several regulators. Many questions about the nature of such codes, however, remain. It is not known, for example, how complex such codes are or how they are established. It is also unclear whether they are confined in their action or if they act outside of their normal context. To address these outstanding issues, we have used two unique subsets of *Drosophila* neurons, identifiable by their specific expression of two different neuropeptide genes. We have identified two partially overlapping and relatively simple codes, consisting of four to seven regulators that act to specify these two cell types. Intriguingly, specification is achieved in a feedforward manner such that A activates B, followed by A/B activating C, and A/B/C activating D. Each code is surprisingly potent, and can ectopically activate neuropeptide gene expression in a variety of neurons, with a surprising disregard for many early patterning events.

## Introduction

Animals have a daunting number of different cell types, and this cellular diversity is profound in the nervous system. During the last two decades, it has become increasingly apparent that neuronal cell fate is not dictated by the action of any single regulatory gene, but results from the combinatorial action of a number of genes that comprise a regulatory “code” [1–3]. Given the enormous diversity of neuronal cell identities, and the great number of regulatory genes in the genome(s), the “decoding” of neuronal cell fate specification is only just beginning.

The appearance of the proper combinatorial code in an early postmitotic neuron is the consequence of a sequence of earlier and increasingly restricted regulatory events [4,5]. How do the earlier regulatory events influence postmitotic cell fate decisions? In one extreme model, early regulators may act simply to ensure proper combinatorial coding in early postmitotic neurons. At another extreme, they may confer a state of competence that to some extent reduces the need for precise and extensive coding of cell fate. If the first model is correct, combinatorial coding should have great informational value and easily traverse many developmental boundaries—once in place, a combinatorial code should be able to act without major restrictions. If the second model is correct, combinatorial codes should have low informational value and be restricted by developmental boundaries—although the proper combinatorial code may be in place, its activity will often be restricted by the history of the cell. Given the extent of neuronal diversity in the mammalian nervous system, and the number of regulatory genes involved, distinguishing between these two models relies on the identification of specific codes in more easily accessible genetic model systems.

**Figure 1:**
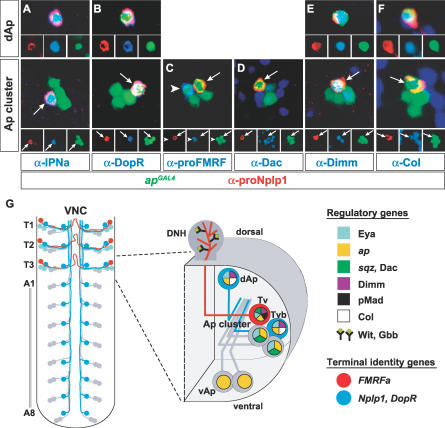
A Specific Subset of Ap Neurons, the Tvb and dAp Cells, Expresses the Nplp1 Neuropeptide, the DopR Dopamine Receptor and the Col Transcription Factor Staining for *ap^GAL4^* (green) and proNplp1 (red), and IPNamide (A), DopR (B), proFMRF (C), Dac (D), Dimm (E), and Col (F) (blue). Arrows denote the Tvb neuron, and arrowheads the Tv neuron. (A and B) Co-expression of proNplp1 with IPNamide (A) and DopR (B) in dAp neurons (top) and one Ap cluster neuron (bottom). (C and D) No co-expression between proNplp1 and proFMRFa (C) or Dac (D). (E and F) Co-expression between proNplp1 and Dimm (E) or Col (F). The Tvb neuron is identified by its expression of *ap,* Eya, Dimm, DopR, and Nplp1, but not Dac or FMRFa. (G) Cartoon summarizes the expression of regulatory genes and terminal identity genes in Ap neurons. DNH, dorsal neurohemal organ; vAp, ventral Ap neurons. All images are from 18-h AEL VNCs.

We are focusing our studies on a specific subset of *Drosophila* ventral nerve cord (VNC) neurons. Within the VNC, only about 90 out of approximately 10,000 cells express the LIM-homeodomain transcription factor Apterous (Ap), but this group of neurons consists of several classes [6] (Figure 1), which are located in three positions in each hemisegment; the single dorsal Ap (dAp) neuron, a ventral pair of (vAp) neurons, and a lateral “Ap cluster” of four neurons found only in each of the six thoracic hemisegments. Most Ap neurons are interneurons that project along a common fascicle. However, in each Ap cluster, the Tv neuron projects its axon out of the VNC at the dorsal midline and innervates the dorsal neurohemal organ (DNH) [7]. Tv neurons uniquely express the FMRFamide (FMRFa) neuropeptide gene [8]. A number of regulatory genes have been found to be important for Tv specification and FMRFa regulation. These include *ap* itself, the zinc finger gene *squeeze (sqz),* the bHLH gene *dimmed (dimm)*, as well as the transcriptional co-factors encoded by the *eyes absent (eya)* and *dachshund (dac)* genes [7,9–12]. In addition, FMRFa expression is completely dependent upon retrograde BMP signaling, specifically mediated by the Glass bottom boat (Gbb) ligand acting on the Wishful thinking (Wit) receptor [9,13] (Figure 1G). Two other *ap* neurons, dAp and Tvb, specifically express the dopamine D1 receptor (DopR) and are also peptidergic [10,14], although the neuropeptide(s) expressed by these neurons was hitherto unknown. Similar to Tv neurons, Tvb/dAp express *ap, dimm,* and *eya,* and all three regulators are necessary for DopR expression in these neurons [14] (this study). This indicates that additional regulators are needed to distinguish Tv neurons from Tvb/dAp neurons.

Here we identify the neuropeptide gene expressed by Tvb/dAp as the *Neuropeptide like precursor protein 1 (Nplp1)* gene. We find that these neurons also express the COE family member *collier (col;* Flybase *knot)*. In *col* mutants, as well as in *ap, eya,* and *dimm* mutants, expression of Nplp1 and DopR is severely affected or completely lost. Genetic analysis further reveals that *col* is expressed in the lineage generating the Ap cluster, and acts at an early postmitotic stage to activate *ap* and *eya,* but not *sqz* and *dac*. *col* subsequently acts with *ap* and *eya* to activate *dimm,* and together with all three regulators to activate the Nplp1 and DopR terminal differentiation genes in a “feedforward cascade.” Combinatorial misexpression of *col, ap, eya,* and *dimm* can potently activate Nplp1 and DopR in many neurons throughout the VNC. Although this code is similar to the FMRFa code, simply replacing *col* with *dac* is sufficient to shift the specificity of the code from Nplp1/DopR expression to widespread activation of FMRFa. In summary, these combinatorial codes are established in a multistep feedforward manner. Once established, they have high informational value for the specification of neuronal identity, and potently activate specific terminal differentiation genes, with limited cross-activation of the incorrect ones. Surprisingly, ectopic activation of terminal differentiation genes can be observed throughout the VNC, disregarding many known developmental boundaries such as anteroposterior, dorsoventral, and mediolateral boundaries.

## Results

### A Subset of Ap Neurons Specifically Express the Neuropeptide Gene *Nplp1* and the Dopamine Receptor DopR

In the developing *Drosophila* VNC, approximately 90 neurons express the LIM-homeodomain regulator Apterous (Ap), and these represent at least six different cell types. Herein, we will focus on three of the Ap neurons: two cells of the Ap cluster—the Tv cells, which express FMRFa, and the Tvb cells, which together with the dAp cells express DopR (Figure 1G). A number of regulators involved in Tv neuron specification have been identified, but to better understand specification of the related Tvb/dAp neurons, we wanted to identify the putative neuropeptide gene expressed by Tvb/dAp neurons. The completion of the *Drosophila* genome led to the prediction of several additional neuropeptide genes, including the *Neuropeptide like precursor protein 1–4* genes (*Nplp1–4*; Flybase, http://flybase.bio.indiana.edu/). The validity of these predictions has been confirmed by the identification of expressed sequence tags (ESTs) matching these genes (Flybase, http://flybase.bio.indiana.edu/), and by the detection of amidated and secreted peptides in circulation, and/or in brain extracts [15]. Expression of gene products from one of these genes, *Nplp1,* was found in a set of cells in the VNC reminiscent of the Tvb/dAp neurons [15]. In situ hybridization for *Nplp1* verified that these cells indeed correspond to the dAp neurons and to one Ap cluster neuron (Figure S1C–S1E). To further identify this Ap cluster neuron, we raised antibodies against pro-Nplp1 and against one of the processed and amidated peptides, IPNamide, and detected a similar pattern (Figure S1A and S1B). We used markers for specific subsets of Ap neurons, and could identify the Nplp1-expressing cell in the Ap cluster as the Tvb neuron (Figure 1A–1E). In Tvb/dAp neurons, Nplp1 and DopR expression commences in the late embryo (18 h after egg laying [AEL]) and persists at least into the third larval stage (unpublished data). Nplp1 and DopR are thus specifically expressed by the 28 embryonic and larval Tvb/dAp neurons (Figure 1G).

### The COE Family Member *col* Is Expressed in Tvb/dAp Neurons and Regulates Nplp1 and DopR

**Figure 2:**
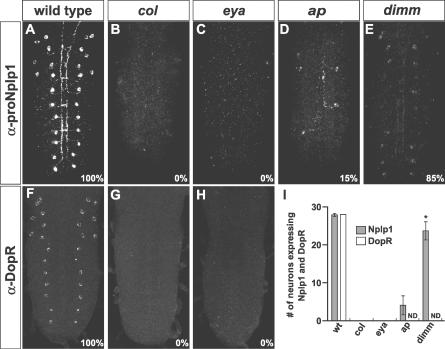
*col* and Other Ap Neuron Determinants Regulate Both Nplp1 and DopR (A–E) Expression of proNplp1 at 18 h AEL in wild type (A), *col* (B), *eya* (C), *ap* (D), and *dimm* (E) reveals a complete loss of expression in *col* and *eya* mutants, and a partial loss in *ap* and *dimm* mutants. (F–H) Expression of DopR in wild-type (F), *col* (G), and *eya* (H) mutants. Expression of DopR is completely lost in *col* and *eya* mutants. (I) Quantification of observed phenotypes (*n* > 10 VNCs). ND (not determined) denotes that DopR expression was not assayed in *ap* and *dimm* mutants because previous studies have already revealed a strong effect of *ap* and *dimm* on DopR expression [14]. The asterisk denotes that Nplp1 is significantly affected in *dimm* mutants (Student *t*-test, *p* < 0.01). wt, wild type. Genotypes: (A and F) *w^1118^*, (B and G) *col^1^/col^3^*, (C and H) *eya^Cli-IID^*/*eya^10^*, (D) *ap^p44^*, and (E) *dimm^p1^*/*dimm^rev4^*.

Recent studies have revealed that *ap* and *dimm* are important for DopR expression in Tvb/dAp [14]. We analyzed whether these Ap neuron determinants, as well as *eya,* also affected Nplp1 expression (Figure 2). We found that Nplp1 expression depended on *eya, ap,* and *dimm* (Figure 2C–2E), and that *eya* also regulates DopR (Figure 2H–2I). How is Tv versus Tvb/dAp cell fate then determined? Although both cell types express *ap, dimm,* and *eya,* only Tv neurons express *dac* and have activated the BMP pathway. Could the mere absence of *dac* and/or BMP activation be sufficient to specify the Tvb/dAp fate? To test this, we analyzed expression of Nplp1 in *dac* and BMP mutants, but found no evidence of ectopic Nplp1 expression in Tv neurons (see below). Thus, specification of Tvb/dAp neurons likely requires additional factors restricted to this cell type.

The COE family of HLH regulators is highly evolutionary conserved [16], and is represented in *Drosophila* by a single member, *col* (Flybase, *knot*) [17]. COE genes play important roles during nervous system development in Caenorhabditis elegans and vertebrates [18–24], and *col* is expressed in the developing *Drosophila* central nervous system (CNS) [17], although no function has yet been assigned to it there. The involvement of members of this gene family in nervous system development in other species, and the embryonic CNS expression of *col,* prompted us to investigate the possible role of *col* during Ap neuron specification. *col* has a dynamic expression pattern in the VNC (below), and we initially focused on its expression in mature Ap neurons, at 18 h AEL, and larval stages. We find that at these stages, *col* is expressed specifically in Tvb/dAp neurons (Figure 1F), and expression is maintained in these neurons at least into the third larval stage (see below). This raised the possibility that *col* plays a role in Tvb/dAp cell fate specification. This notion was supported by the complete loss of Nplp1 and DopR expression in *col* mutants (Figure 2A, 2B, 2F, 2G, and 2I).

### *col* Acts Upstream of Certain *ap* Neuron Determinants

Previous studies have addressed the regulatory interactions between several of the Ap neuron determinants [9–12]. However, these had not been addressed in the case of *dac, eya,* and *dimm*. As expected from the late onset of *dimm* expression, we find no evidence of *dimm* regulation of Dac or Eya (Figure S2M and S2N). Similarly, as expected from the mild effect of *dac* upon FMRFa, we find that *dac* does not regulate Dimm (Figure S2L). In contrast, in *eya* mutants, we find a nearly complete loss of Dimm expression in the Tv, Tvb, and dAp neurons (Figure S2K and S2O).

With a more complete picture of how previously identified Ap neuron determinants interact genetically, we next addressed whether *col* acts upstream, downstream, or in parallel to other Ap neuron determinants. We first analyzed the expression of Col in embryos mutant for these other regulators. In general, we found no severe effects on Col expression (Figures S2A–S2I). The one exception was in *sqz,* in which we found a reproducible increase in the number of Col cells (Figure S2B, S2C, and S2J). This was, however, expected, since *sqz* affects the composition of Ap cluster cells, with an increase both in the number of Ap cluster cells (specifically in T1) and an increase in Tvb cells at the expense of Tv cells (in T1–T3) [9,10]. In line with this, we also find an increase in Nplp1 cells in *sqz* mutants (Figure S2B, S2C, and S2J).

**Figure 3:**
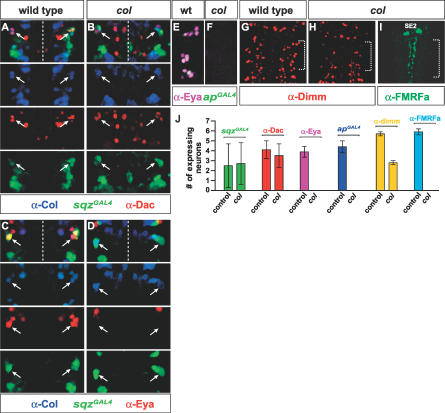
*col* Regulates *ap, eya, dimm,* and FMRFa, but Not *sqz* and *dac* (A–I) Expression of Ap neuron determinants and of FMRFa (I), in wild-type and *col* mutants at stage 15 (A–D) and 18 h AEL (E–I). Dashed lines in (A–D) depict the VNC midline. (A and C) In wild type, expression of Col (blue), *sqz^GAL4^* (green), Dac (red), and Eya (red) clearly visualizes the early Ap cluster (arrows). (B and D) In *col* mutants, although *sqz^GAL4^*and Dac expression is largely unaffected (B), there is a complete loss of Eya expression in the Ap cluster (D, arrows). (E and F) In the late embryonic VNC, Eya and *ap^GAL4^* are readily detected in all three thoracic hemisegments in the wild type (E), whereas *col* mutants show complete loss of expression of both markers (F). (G and H) Similarly, Dimm expression (red) is lost from lateral thoracic peptidergic neurons in *col* mutants (bracket). Staining for other neuropeptides such as CCAP and Corazonin reveals that Dimm expression is specifically lost from Tv, Tvb, and dAp peptidergic neurons (unpublished data). (I) As expected from the loss of several Ap neuron determinants, FMRFa expression (green) is completely lost from Tv neurons (bracket). As with other mutations specifically affecting Tv neurons, expression of FMRFa is not lost from the SE2 neurons (SE2). (J) Quantification of the observed phenotypes (for *sqz^GAL4^,* Dac, Eya, and *ap^GAL4^, n* > 65 clusters; for Dimm, *n* > 65 thoracic hemisegments; and for FMRFa, *n* > 24 VNCs). *sqz^GAL4^,* Dac, and Eya are quantified at stage 15, whereas *ap^GAL4^,* Dimm, and FMRFa are quantified at 18 h AEL. Genotypes: (A and C) *UAS-nmEGFP/+; sqz^GAL4^*/*+,* (B and D) *col^1^, UAS-nmEGFP*/*col ^3^ sqz^GAL4^/+,* (E) *ap^GAL4^/+; UAS-nmEGFP/+,* (F) *col^1^, ap^GAL4^*/*col^3^; UAS-nmEGFP/+,* (G) *w^1118^,* and (H and I) *col^1^/col^3^*. (J) Controls are: *UAS-nmEGFP/+; sqz^GAL4^/+* for *sqz,* Dac, and Eya expression; *ap^GAL4^/+; UAS-nmEGFP/+* for *ap* expression; and *w^1118^* for Dimm and FMRFa expression.

Does *col* act upstream of other Ap neuron determinants instead? To address this, we analyzed the expression of these other regulators in *col* mutant embryos. This analysis was facilitated by the fact that *col^2^* and *col^3^* mutants, which are both genetically strong alleles, are not protein null, thus allowing for detection of Col in *col* mutants. In addition, Col is expressed by all four Ap cluster neurons at stage 15 (see below). In *col* mutants (Figure 3), we find that although *sqz* and Dac are largely unaffected (Figure 3A, 3B, and 3J), *ap,* Eya, and Dimm are all completely absent from Ap neurons (Figure 3C–3H and 3J). Because *ap, eya,* and *dimm* all regulate FMRFa, the loss of these regulators prompted us to look at the expression of FMRFa as well, and as expected, in *col* mutants, we find a complete loss of FMRFa in the Tv neurons (Figure 3I and 3J). However, FMRFa is still expressed in the SE2 neurons, a feature common to all identified FMRFa regulators except *dimm* (Figure 3I). These results suggest that Ap cluster neurons are generated in *col* mutants, but are incompletely specified because they express part of their normal specification code such as *dac* and *sqz,* but not other elements of the code such as *ap,* Eya, Dimm, FMRFa, Nplp1, and DopR.

### The Ap Cluster Is Generated by Thoracic Neuroblast 5–6 and *col* Is Expressed at a Late Stage in This Lineage

The severe effect of *col* upon *ap, eya,* and *dimm* within all Ap cluster neurons, and upon FMRFa within the Tv neuron, is at odds with the restricted expression of Col in Tvb/dAp neurons at late embryonic stages. We therefore analyzed whether Col is more widely expressed at earlier embryonic stages, focusing on the Ap cluster neurons. This revealed that although *col* is restricted to Tvb neurons at stages 17 and 16 (Figure S3K and S3L), we observe expression in all four Ap cluster neurons at stage 15, the stage at which these neurons are first identifiable using *ap,* Eya, Dac, and *sqz* as specific markers (Figure S3E).

Is Col expressed even in the progenitor cells generating Ap cluster neurons? Because *ap,* Eya, Dac, and *sqz* are not expressed in Ap cluster neurons prior to stage 15, resolving this issue required the identification of the neuroblast lineage generating the Ap cluster. Extensive work during the last two decades has provided a detailed lineage map of most, if not all, of the 30 neuroblasts found in each hemisegment, and has identified regulatory genes expressed by different neuroblasts [25–29]. These studies, together with the extreme lateral positioning of early Ap cluster neurons and their appearance only in thoracic hemisegments, allowed us to use a series of specific markers and determine that the Ap cluster is generated by NB 5–6—a lateral-most neuroblast that has been shown to generate larger lineages in the thoracic segments [28,29] (see Figure S3 and Text S1 for a full description of the mapping of Col expression in NB 5–6). These results, combined with previous detailed studies of NB 5–6 development, allow us to outline a tentative model for the thoracic NB 5–6 lineage, and to place Col expression within this lineage (see below).

### Down-Regulation of *col* Is Critical for Proper *ap* Cluster Differentiation

**Figure 4:**
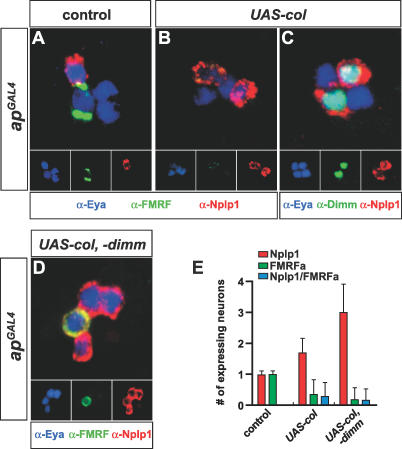
*col* Misexpression in the Ap Cluster Triggers Ectopic Nplp1 Expression (A) In the control, the Ap cluster (here visualized by Eya) contains one FMRFa- and one Nplp1-expressing cell. (B and C) Misexpression of *col* in the Ap cluster triggers ectopic Nplp1 expression in one additional Ap cluster neuron. Labeling for FMRFa (B) and for Dimm (C) reveals that this cell is the Tv neuron, evident from its co-expression of Nplp1, FMRFa (B), and Dimm (C). (D) Co-misexpression of *col* and *dimm* in the Ap cluster triggers all four Ap cluster neurons to express Nplp1. Misexpression of *dimm* has no effect upon Nplp1 expression (unpublished data). (E) Quantification of Nplp1, FMRFa, and Nplp1/FMRFa expression (*n* > 80 thoracic hemisegments). All images are from 18-h AEL VNCs. Genotypes: (A) *w^1118^,* (B and C) *ap^GAL^* ^4^ */+; UAS-col/+,* and (D) *ap^GAL4^/+; UAS-col, UAS-dimm*/*+*. (E) Control is *w^1118^*.

Col is expressed by all four newly born Ap cluster neurons and is essential for Ap cluster specification, as evident from the complete loss of *ap* and Eya expression in *col* mutants. Col is rapidly down-regulated from three Ap cluster cells and maintained only in Tvb. Is the down-regulation of *col* important for proper Ap cluster differentiation? To test this, we misexpressed *col* using the *ap^GAL4^* driver, which is not expressed until stage 16, thus maintaining col expression in all four Ap cluster neurons at the time when Col is normally down-regulated. This experiment led to frequent activation of Nplp1 in one additional Ap cluster neuron (Figure 4A and 4B), and staining for Dimm reveals that this cell is indeed the Tv neuron (Figure 4C). FMRFa expression is frequently down-regulated in Tv, but we do observe Tv cells that co-express FMRFa and Nplp1 (Figure 4B). The finding that *col* misexpression in the Ap cluster only leads to one ectopic Nplp1 cell and no ectopic Dimm cells indicates that *col* cannot induce a peptidergic cell fate, at least not with this late driver. However, because the two unaffected cells already are expressing *ap* and Eya, we predicted that co-misexpression of *dimm,* together with *col,* should trigger Nplp1 expression in all four Ap cluster neurons. This is indeed what we find (Figure 4D and 4E). In summary, down-regulation of *col* in three Ap cluster neurons is essential for proper Ap cluster specification.

### *col* Can Act in a Context-Dependent Manner to Generate Additional Ap Cluster Neurons

**Figure 5:**
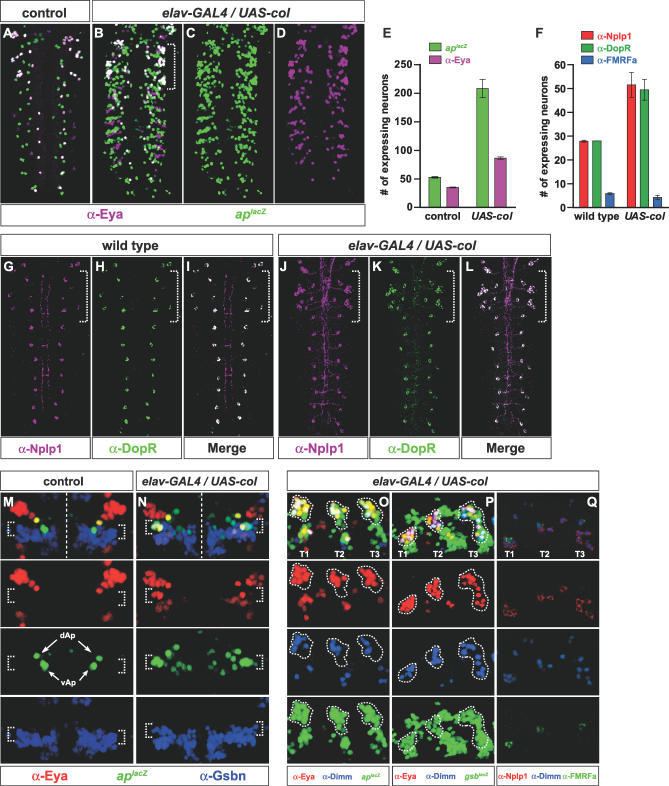
Misexpression of *col* Activates *ap,* Eya, Dimm, Nplp1, and DopR, but in a Context-Dependent Manner (A–L) Expression of *ap^lacZ^,* Eya, Nplp1, DopR, and FMRFa in 18-h AEL control and *col* misexpression VNCs. (A) In the control, *ap^lacZ^* and Eya are specifically expressed in Ap neurons. A small number of additional Eya-expressing cells are visible laterally; these cells express Eya at earlier stages, but typically down-regulate Eya expression at stages 15/16 [12]. (B–D) In *col* misexpression embryos, robust ectopic expression is evident for both *ap^lacZ^* and Eya, although ectopic co-activation of *ap^lacZ^* and Eya genes is predominantly apparent in lateral thoracic areas (bracket). (E) Quantification of *ap^lacZ^* and Eya expression (*n* > 3 VNCs). (F) Quantification of Nplp1, DopR, and FMRFa expression (*n* > 8 VNCs). There is no evidence of ectopic FMRFa expression. (G–I) In the wild type, Nplp1 and DopR expression is evident in dAp and Tvb neurons. (J–L) *col* misexpression leads to ectopic co-activation of Nplp1 and DopR expression, again restricted to lateral thoracic areas (brackets). (M and N) *col* misexpression triggers ectopic co-activation of *ap^lacZ^* and Eya at stages earlier than when Ap clusters are normally generated (dashed lines depict the VNC midline). In the control (M), only dAp (*ap^lacZ^*/Eya expressing) and vAp (*ap^lacZ^* expressing) neurons are visible at stage 14. (N) In *col* misexpression, ectopic *ap^lacZ^* and Eya cells, as well as double-positive cells, appear earlier and increase in numbers into stage 14 (brackets). (O–Q) Lateral view of the three thoracic segments in 18-h AEL VNCs; anterior is to the left. (Dotted circles depict the enlarged Ap cluster.) (O) *col* misexpression leads to appearance of six to ten *ap^lacZ^*/Eya-expressing cells, of which a subset is also Dimm expressing, and (P) these ectopic “Ap cluster” cells are confined to row 5, i.e., the anterior region of *gsb^lacZ^* expression. (Q) *col* misexpression triggers ectopic Nplp1 in these ectopic Dimm cells, while FMRFa is still only expressed in one cell per hemisegment. Genotypes: (A and M) *ap^lacZ^/+; elav-GAL4/+,*  (B–D, N, and O) *ap^lacZ^/+; elav-GAL4/ UAS-col/+,* (G–I) *w^1118^,* (J–L) *elav-GAL4/UAS-col,* and (P–Q) *gsb^lacZ^/+; elav-GAL4/ UAS-col/+.* (E) Control is *ap^lacZ^/*+; *elav-GAL4/+,* and (F) control is *w^1118^*.

Col is expressed prior to *ap* and Eya in the Ap cluster neurons, and it is essential for *ap* and Eya expression within these cells. To address whether *col* is also sufficient to activate *ap* and Eya, we misexpressed *col* in all neurons, using the *elav-GAL4* driver (Figure 5) [30]. This led to ectopic activation of both *ap* and Eya (Figure 5A–5E). In addition, we found some activation of Dimm, Nplp1, and DopR expression (Figure 5F–5L and 5O–5Q). Although ectopic activation of *ap* or Eya alone was found in several regions, co-activation was largely confined to neuroblast row 5—the anterior region of Gsbn expression (Figure 5M and 5N). We typically observe six to ten *ap*/Eya co-expressing cells in the lateral-most part of row 5 (Figure 5M and 5N). Ectopic activation of *ap*/Eya, together with Dimm, Nplp1, and DopR, was also confined to lateral-most row 5, i.e., the posterior-most part of *gsb^lacZ^* cells, and further confined to thoracic segments (Figure 5B–5D, 5J, 5L, and 5O–5Q). Ectopic Nplp1/DopR expression is not overlapping with FMRFa, and there is clear evidence of ectopic Dimm expression (Figure 5P and 5Q), indicating that additional peptidergic neurons are being generated. Ectopic generation of *ap*/Eya double-expressing cells, i.e., ectopic “Ap cluster” neurons, was observed already at stage 13, i.e., prior to when Ap cluster neurons are normally born (unpublished data).

These results show that *col* can activate *ap* and Eya in a number of neurons, but can act to generate bona fide Ap cluster neurons only in a highly context-dependent manner: in lateral, thoracic, row 5 neurons. The appearance of six to ten Eya-expressing cells, but only three to five Nplp1/DopR-expressing cells, and no evidence of ectopic FMRFa expression, suggests that the generation of ectopic Ap cluster neurons is biased toward Tvb (Nplp1/DopR expressing) as opposed to Tv (FMRFa expressing) cell fate. In contrast, although *col* function depends upon these three positional cues, our results indicate that *col* is able to override the temporal coding within lateral row 5, and activate Nplp1 and DopR in earlier-born neurons.

### *col* Can Be Partially Rescued by Expression of *ap* and *eya*

**Figure 6:**
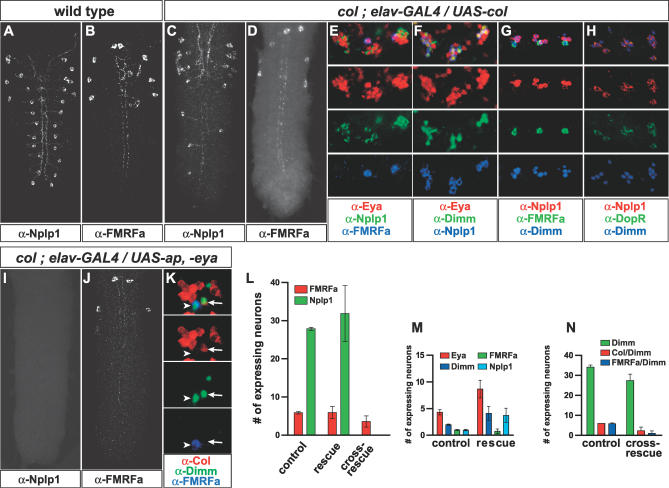
*col* Rescues Both Tv and Tvb Cell Fate in *col* Mutants, whereas *ap* and *eya* Only Rescue Tv Cell Fate (A and B) Wild-type expression of Nplp1 and FMRFa. (C–H) *col* rescue of *col* mutants, in 18-h AEL VNCs. (C and D) *col* expression in all neurons efficiently rescues back both Nplp1 and FMRFa expression in *col* mutants. (E–H) Similar to the misexpression results (Figure 5), *col* rescue results in generation of extra “Ap cluster” neurons, as evident from the expression of Eya, Dimm, Nplp1, and DopR, whereas there is no evidence of ectopic FMRFa expression (lateral view of three thoracic VNC hemisegments; anterior is to the left). (I–K) “Cross-rescue” of *col* with *ap* and *eya* leads to a significant rescue of FMRFa (*p* < 0.01, *n* = 35 VNCs), but no rescue of Nplp1. (K) “Cross-rescue” does lead to a partial rescue of Dimm expression in cells expressing Col (a putative “Tvb” cell; arrow), but this cell is never able to activate Nplp1. (The arrowhead points to the FMRFa-expressing Tv cell.) Col can be visualized because the *col^3^* allele is not a protein null. (L–N) Quantification of observed phenotypes (*n* > 16 VNCs for [L], *n* > 41 hemisegments for [M], and *n* > 9 hemisegments for [N]). (L) Expressing neurons per VNC. (M) Expressing neurons per Ap cluster. (N) Expressing neurons for the three thoracic segments combined. Genotypes: (A and B) wild type, (C–H) *col^1^/col^3^; elav-GAL4*/*UAS-col,* and (I–K) *col^1^/col^3^; elav-GAL4*/*UAS-ap, UAS-eya*. (L–N) Controls are *w^1118^*.

The loss- and gain-of-function studies place *col* clearly upstream of *ap* and *eya*. Does *col* act merely to regulate *ap* and *eya* in early postmitotic Ap cluster neurons, or does it play additional roles during Ap cluster formation? To further address this issue, we attempted to “cross-rescue” *col* with *ap* and *eya,* by expressing *ap* and *eya* in a *col* mutant background (Figure 6). First, as a positive control, we attempted to rescue *col* by providing *col* activity using *elav-GAL4/UAS-col*. This led to a robust rescue, both of Ap cluster determinants (Eya, *ap,* and Dimm) and of terminal differentiation genes *(Nplp1, DopR,* and *FMRFa)* (Figure 6A–6H, 6L, and 6M). Similar to the *col* misexpression experiments, we find a clear increase in “Ap cluster” neurons, primarily of the Tvb type, as evident from the finding of six to ten *ap*/Eya- and three to five Nplp1/DopR-expressing neurons per hemisegment (Figure 6E–6H and 6M). Next, we attempted to “cross-rescue” *col* mutants with *ap* and *eya,* and could indeed find a significant degree of rescue of Ap cluster formation, as evident both from Dimm and FMRFa expression (Figure 6J–6M). In contrast, we found no evidence of rescue of Nplp1 or DopR in these embryos (Figure 6I and 6L). Because we can detect Col in the *col^2^* and *col^3^* mutant backgrounds, we could identify a Dimm/Col-expressing cell adjacent to the Tv/FMRFa neuron (Figure 6K and 6N). This indicates that *ap/eya* can partially rescue Tvb cell fate, but in the absence of *col* activity, these “Tvb” neurons do not activate Nplp1. In summary, the finding that in *col* mutants, *ap* and *eya* can partially rescue the Tv cell fate, but not Tvb cell fate, suggests additional roles for *col* in Tvb specification.

### *col* Acts at Multiple Steps of Tvb Specification

**Figure 7:**
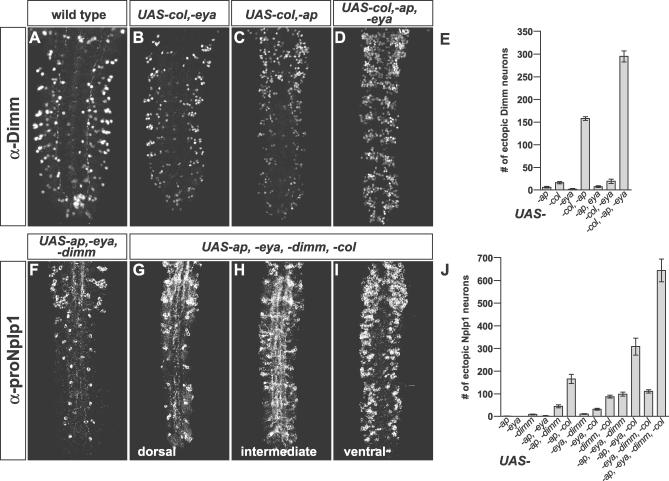
*col* Can Act in a Combinatorial Manner with *ap* and *eya* to Activate Dimm, and with All Three Regulators to Activate Nplp1 and DopR (A–E) Expression of Dimm in 18-h AEL VNCs. Single misexpression of each regulator has limited effect upon Dimm expression, but combinatorial expression of *col/ap* or *col/ap/eya* leads to striking ectopic Dimm expression. (E) Quantification of Dimm expression in the various misexpression experiments (*n* > 3 thoracic T1–T3 segments). Importantly, the addition of *col* to the different codes has greatly increased effect upon Dimm—compare *ap/eya* with *col/ap/eya*. (F–J) Expression of Nplp1 in 18-h AEL VNCs. Single misexpression has limited effect upon Nplp1 expression, but combinatorial expression leads to strong ectopic Nplp1 expression. (J) Quantification of Nplp1 expression (*n* > 3 VNCs; numbers denote total number of expressing cells per VNC). Importantly, the addition of *col* to the different codes has greatly increased effect also upon Nplp1—compare *ap/eya/dimm* with *ap/eya/dimm/col*. Genotypes: (A) *w^1118^* and (B–J) *elav-GAL4* crossed to single *UAS-cDNA* transgenes or combinations of the various *UAS-cDNA* transgenes.

Our results indicate that Tvb cell fate is not specified by a linear *col*→*ap/eya*→*dimm*→*Nplp1/DopR* genetic cascade. To further address this issue, we tested the sufficiency of *col, ap,* and *eya* to activate Dimm when misexpressed both alone and in combination. These experiments reveal that although *col* can trigger some ectopic activation of Dimm, there is little effect upon Dimm when misexpressing *ap, eya,* or *ap/eya* (Figure 7A). In contrast, co-misexpression of *col* with either *ap* or *eya,* and in particular, co-misexpression of all three genes, leads to striking ectopic Dimm expression (Figure 7A–7E).

Does *col* play a role even at the final step of Tvb differentiation, i.e., in the activation of Nplp1? We attempted to address the possible late role of *col* by misexpressing it alone and together with other Ap neuron determinants, and then assay its potency in activating Nplp1. Importantly, if there is a simple linear *col*→*ap/eya*→*dimm* *Nplp1/DopR* genetic cascade at work, the effect of triple co-misexpression of *ap/eya/dimm* should not be enhanced by addition of *col* to this code. However, we find a striking enhancement of ectopic Nplp1 expression when adding *col* to this code (Figure 7F–7J). One particular double co-misexpression combination, *col*/*ap,* was more potent than others in activating both Dimm and Nplp1 (Figure 7E and 7J). A likely explanation for this effect is that co-misexpression of *col*/*ap* activates significant ectopic *eya* expression (unpublished data).

**Figure 8:**
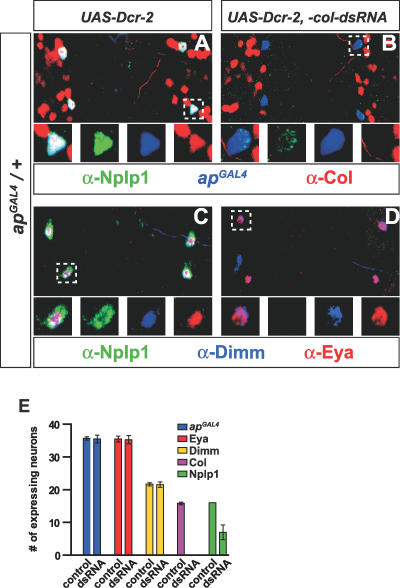
Postmitotic RNA Interference of *col* Leads to Loss of Nplp1 Expression, without Loss of *ap,* Eya, or Dimm (A and C) In the control, expression of Nplp1, *ap,* and Col (A), as well as Eya and Dimm (C) is readily detected in the third larval instar dAp neurons. (B and D) In the *col* RNAi, Col is completely and specifically lost from dAp (B), Nplp1 is down-regulated (B) or lost (D), whereas *ap,* Eya, and Dimm are not affected (B and D). As anticipated, *col* RNAi has no effect upon FMRFa expression in the third instar larvae (*n* = 5 VNCs; unpublished data). Dashed boxes outline the cells that are magnified at the bottom. (E) Quantification of the observed effects in dAp and Ap cluster neurons, in segments T1–A2. Genotypes: (A and C) and control in (E) *UAS-Dcr-2/+; ap^GAL4^, UAS-nmEGFP/+* and (B and D) *UAS-Dcr-2/+; ap^GAL4^, UAS-nmEGFP/+; UAS-col-dsRNA/+.*

To further address the late role of *col,* we generated a transgenic RNA-interference (RNAi) line *(UAS-col-dsRNA)* and attempted to suppress *col* gene activity by crossing this line to *ap^GAL4^*. Because *ap^GAL4^* also drives expression in the developing wing disc, we first analyzed the efficiency of this novel tool in suppressing *col* gene activity in this tissue. This phenocopied the effects of *col* mutants on wing development [31], with a clear L3–L4 wing vein fusion (unpublished data), indicating that this RNAi transgene specifically blocks *col* gene activity. However, upon analyzing late larval (third instar) CNSs, we found no effect upon Col expression in Tvb neurons, and as expected, no effect upon Nplp1 expression (unpublished data). Recent studies reveal that RNAi can be efficiently enhanced by overexpression of components of the RNAi pathway, in particular of the *Dicer-2 (Dcr-2)* gene (G. Dietzl and B. Dickson, personal communication). We therefore co-expressed *Dcr-2* with *col* dsRNA *(UAS-Dcr-2*/*+; ap^GAL4^*/*UAS-col-dsRNA),* and found a clear effect not only upon Col, but importantly, also upon Nplp1 expression. We found no obvious effect in the first instar larvae (unpublished data), but in third instar larvae, Col expression is specifically and completely lost from all Tvb/dAp cells (Figures 8A, 8B, and 8E). This leads to a complete loss of Nplp1 in 44% of Tvb/dAp cells (Figure 8C–8E), and strongly reduced expression in the remaining expressing cells (Figure 8B). Strikingly, this strong effect upon Nplp1 is not an indirect effect of down-regulation of *ap,* Eya, or Dimm (Figure 8A–8E). As anticipated, *col* RNAi has no effect upon FMRFa expression in the third instar larvae (*n* = 5 VNC; unpublished data).

Together, the finding that *col* can act potently with *ap* and *eya* to activate Dimm, that *col* can act potently with *ap, eya,* and *dimm* to activate Nplp1, and that postmitotic RNAi of *col* strongly affects Nplp1 expression without affecting *ap,* Eya, or Dimm, does not fit with a simple linear cascade model for Tvb specification. Rather, it strongly suggests that *col* is acting at several levels during Tvb specification and differentiation.

### The Combinatorial Codes Are Highly Potent and Highly Specific

**Figure 9:**
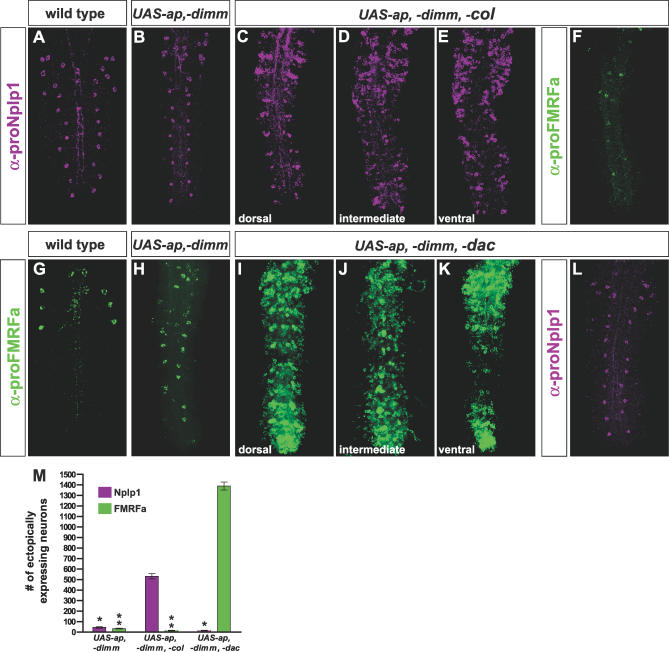
The Two Combinatorial Codes Act in a Highly Potent and Highly Selective Manner Expression of Nplp1 (A–E and L) and FMRFa (F–K). (A) Wild-type expression of Nplp1. (B) Co-misexpression of *ap* and *dimm* leads to some ectopic Nplp1 expression. (C–E) Ectopic Nplp1 expression is dramatically increased by addition of *col* to this code (to visualize the strong activation, the confocal “stack” of the VNC is split into three non-overlapping merged images). (F) The same VNC as in (C–E) revealing limited ectopic activation of FMRFa. (G) Wild-type expression of FMRFa. (H) Co-misexpression of *ap* and *dimm* also leads to some ectopic FMRFa expression. (I–K) Ectopic FMRFa expression is dramatically increased by addition of *dac* to this code. (L) The same VNC as in (I–K) revealing limited ectopic activation of Nplp1. (M) Quantification of the ectopic activation effects (*n* > 3 VNCs). A single asterisk denotes that the ectopic activation of Nplp1 is significantly reduced when adding *dac* to *ap/dimm* (Student *t*-test, *p* < 0.01). Similarly, double asterisks denote that the ectopic activation of FMRFa is significantly reduced when adding *col* to *ap/dimm* (Student *t*-test, *p* < 0.01). All images are from 18-h AEL VNCs. Genotypes: (A and G) *w^1118^*. Remaining panels are *elav-GAL4* crossed to single or combinations of the various *UAS-cDNA* transgenes.

Previous studies have identified several regulators acting to specify Tv fate and to control FMRFa expression. Although co-misexpression of parts of this code had been previously tested, all possible combinations had not. Similar to the combinatorial activation of Nplp1 and DopR, we find that whereas co-misexpression of *ap/sqz, ap/dac,* or *ap/dimm* has limited effect upon FMRFa expression (Figure 9) [9,10,12], triple co-misexpression of these regulators, and in particular of *ap/dimm/dac,* leads to a dramatic ectopic activation of FMRFa (Figure 9G–9K and 9M).

The identification of two partly overlapping and highly potent combinatorial codes allowed us to ask an important question: Does combinatorial misexpression of these regulators merely lead to a general confusion with a mixed neuronal identity, or are these codes truly instructive and specific? To address this issue, we analyzed the expression of Nplp1 and FMRFa in the various misexpression backgrounds. Not surprisingly, when common and partial components of these codes are misexpressed, such as *ap/dimm* (common to both Tv and Tvb/dAp neurons), we find ectopic activation of both Nplp1 and FMRFa in different subsets of cells (Figure 9B, 9H, and 9M). However, as a third, and cell-type specific, regulator is added, not only does the amount of ectopic, terminal differentiation gene expression increase dramatically, but we find less evidence of cross-activation of the inappropriate downstream gene (Figure 9C–9F and 9I–9M). This surprising finding reveals that combinatorial misexpression may act in a highly specific and instructive manner, and that these combinatorial codes may be viewed as potent binary switches for cell fate specification. In addition, for both codes, ectopic activation of FMRFa and Nplp1 is observed in neurons throughout the VNC and brain, and traverses many developmental boundaries, such as anteroposterior, dorsoventral, and mediolateral boundaries.

## Discussion

**Figure 10:**
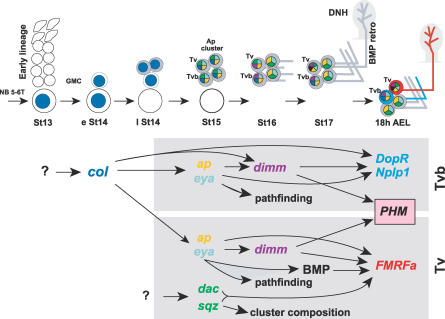
Model for the Thoracic NB 5–6 Lineage and for the Genetic Pathways Specifying Tv and Tvb Neurons The thoracic NB 5–6 lineage is shown at top, and the genetic pathways specifying Tv and Tvb neurons are at the bottom. NB 5–6 is formed already at late stage 8 and generates an early lineage of neurons (circles) and glia (uneven shape). For clarity, the early lineage is omitted from stage 14 and onward. The Ap cluster is generated between stages 13–15. The fate of the neuroblast itself is unclear after stage 15. Some of these regulatory interactions are occurring also in the non-peptidergic Ap cluster neurons, as well as in the dAp neurons, but for simplicity, they are not outlined here. See text for details. DNH, dorsal neurohemal organ; St, stage.

We have identified a sequential regulatory cascade of combinatorial coding that acts to specify two unique neuronal cell fates during *Drosophila* CNS development. Combined with previous studies [6,7,9–14], the findings described in this study provide the following model for Ap cluster generation and specification (Figure 10). Neuroblast 5–6 forms in the first wave of neuroblast delamination, at late stage 8, and generates a mixed lineage of glia and neurons [28,29]. At stage 13, Col expression is turned on specifically in thoracic NB 5–6, in two subsequent ganglion mother cells (GMCs) at stages 13/14, and in the four Ap cluster neurons generated from these GMCs, during stages 14/15. We have not resolved the birth order of the four Ap cluster neurons, and the sibling relationship of Tv and Tvb is thus unclear. When Ap neurons are born, *col* activates *ap* and *eya,* whereas *sqz* and *dac* are activated by unknown regulator(s). *sqz* appears to play an early postmitotic role, apparently acting in the Notch pathway, to ensure proper Ap cluster composition, and *sqz* mutants display both additional Ap cluster cells (in T1) and additional Tvb cells (in T1–T3). *col* is rapidly down-regulated from three Ap neurons, but remains expressed in the Tvb, where it acts with *ap* and *eya* to activate *dimm* at stage 16. At late embryogenesis, *col* acts with *ap, eya,* and *dimm* to activate Nplp1 and DopR in Tvb. In the Tv neuron, *ap* and *eya* act, apparently independently of *col,* to activate *dimm* expression. In the Tv neuron, *eya* furthermore plays a role in setting up competence to respond to the BMP signal. At stage 17, the Tv axon reaches the dorsal neurohemal organ (DNH) and receives the BMP ligand Gbb that activates the Wit receptor, and then triggers activation of the BMP pathway in the Tv neuron. At 18 h AEL, *ap, eya, sqz, dac, dimm,* and BMP signaling cooperate to activate FMRFa in the Tv neuron. In addition to their roles in neuropeptide regulation and BMP signaling, both *ap* and *eya* act to ensure proper axon pathfinding of Tv neurons *(eya),* as well as Tvb and dAp neurons *(ap* and *eya)*. The role that *col* may play more directly in axon pathfinding has not been resolved due to the fact that the expression of the appropriate axonal markers (*ap^GAL4^*, Nplp1, DopR, and FMRFa) is completely absent in *col* mutants. Given the complexity of axon pathfinding, we would anticipate that several other regulators are yet to be identified before a combinatorial code for “Tv-type” or “Tvb-type” axonal pathfinding is deciphered. Indeed, we find no evidence that combinatorial misexpression of the abovementioned regulators can dictate axonal projections, because ectopic Nplp1 or FMRFa axons are following many different routes in the VNC (Figures 7H and 9I). Finally, *dimm* also plays additional roles to those described above and is necessary for the expression of neuropeptide-processing enzymes in peptidergic neurons. Importantly, *dimm* acts independently to control expression of the neuroamidase gene *PHM* in the Tv and Tvb neurons, and *dimm* is sufficient to activate *PHM* in most, if not all, VNC neurons. Thus, during the specification and differentiation of the Ap neurons, there exists a remarkable diversity in the division of labor between the identified regulators, with most of them participating in more than one, but never all, of the identified events.

### Sequential Combinatorial Coding: Feedforward Loops Providing High-Fidelity Control of Neuronal Specification

We have identified a multistep process for specifying the Tv and Tvb cell fates. What would be the purpose of this type of sequential combinatorial coding? In other model systems with higher genetic resolution, such as Escherichia coli and yeast, extensive genetic analysis has revealed that this type of sequential gene regulation is quite common [32,33], and a recent study in C. elegans suggests it may also function during neuronal specification [34]. These regulatory nodes, also known as feedforward loops (FFL), have been shown to ensure fidelity in gene regulation [35,36]. For instance, in a simple FFL in which gene A regulates gene B, and A/B then co-operate to regulate C, activation of C depends upon prolonged A expression such that A/B will have time to activate C. If A is only active in a short burst, B may be activated, but C is not, because A/B never co-express for a sufficiently prolonged period of time. The role of *col* during Ap cluster specification provides an excellent example of a FFL used during neuronal cell fate specification. *col* is expressed in all four Ap cluster cells and plays an early role in activating *ap* and Eya, but is only maintained in Tvb, where it plays a later role in activating first *dimm,* then Nplp1. Maintained expression of *col* in all four Ap cluster neurons, by driving *col* expression from *ap^GAL4^,* leads to activation of the Tvb program also in the Tv neuron. Thus, a burst of *col* expression has a different informational value than persistent *col* expression—general Ap cluster specification versus Tvb specification.

### Combinatorial Coding and Global Cues: Context Dependence versus Independence

Misexpression of each of the two identified combinatorial codes leads to striking ectopic activation of the Nplp1 and FMRFa genes, and we are surprised by two particular aspects of these findings. First, the global potency of these codes—co-misexpression triggers ectopic FMRFa of Nplp1 in a number of neurons, located in many different anteroposterior, dorsoventral, and mediolateral positions. Thus, it would appear that early regulators mainly act to ensure proper combinatorial coding in each neuron, and play a minor role in restricting cell fate by limiting the cell's competence—once the proper code is in place, the cell fate specification program will be carried out irrespective of the history of the cell. Second, the striking binary effect of these codes is noteworthy—the change of one single player in a code completely alters target gene choice. For instance, misexpression of *ap/dimm/dac* leads almost exclusively to strong FMRFa activation, but simply replacing *dac* with *col* leads to almost exclusively Nplp1 activation. Thus, it appears that more complete codes not only have great potency, but also have great specificity.

### The Maintenance of Col, Ap, and Eya Expression

Col shows a very dynamic expression pattern in the VNC—exemplified in NB 5–6 by the expression in the neuroblast, in two GMCs, in all four Ap cluster neurons, and finally only in Tvb. This poses three obvious questions: what activates *col* in the neuroblast, what shuts it down in three of the Ap cluster neurons, and finally, what maintains *col* in Tvb? As for the activation of *col* in the late 5–6 neuroblast, row 5 neuroblast determinants, thoracic determinants, and late temporal determinants are obvious candidates. Indeed, our current work has identified input from a number of such upstream regulators (D. Karlsson, M. Baumgardt, and S. Thor, unpublished data). It is less clear why *col* expression is lost from three Ap cluster cells and maintained in Tvb. It is possible that the initial expression in all four Ap cluster cells merely reflects residual expression, as an effect of the activation by earlier determinants acting in the neuroblast. But why is *col* then maintained in Tvb, and similarly, what maintains *eya* and *ap* in all four Ap cluster cells? One simple solution would be autoregulation of each gene. But surprisingly, there is no evidence of autoregulation of *col* (this study) or *ap* [6,9] (*eya* has not been addressed). In addition, we find no clear evidence of cross-regulation between *col, ap,* or *eya,* at least not during embryonic stages. Thus, it seems likely that other mechanisms, either unidentified regulators or, perhaps, epigenetic mechanisms, act to ensure the continual expression of these regulators during larval (and perhaps adult) life.

## Materials and Methods

### Fly stocks.

The following stocks were used: *ap^md544^* (referred to as *ap^GAL4^*), *ap^P44^, ap^rK568^* (referred to as *ap^lacZ^*), *sqz^DF^, sqz^GAL4^, sqz^ie^, UAS-sqz, UAS-ap, wit^A12^, wit^B11^, UAS-myc-EGFP–farnesylation* (referred to as *UAS-mEGFP^F^*) [9]; *dimm^p1^*, *dimm^rev4^, UAS-dimm* [11]; *elav-GAL4,* [30]; *dac^3^, dac^4^* [37]; *UAS-dac* [38]; *eya^Cli-IID^* [39]; *Df(2L)eya10* (referred to as *eya^10^*), *P{UAS-eya.B.II}* (referred to as *UAS-eya*) [40]; *UAS-nls-myc-EGFP* (referred to as *UAS-nmEGFP*) [41]; *ladybird* fragment K driving *lacZ* (referred to as *lb-lacZ*) [42]; *col^1^, col^2^, col^3^* [43]; *UAS-col* [31]; *P{lacZ-un4}hkb^5953^* (referred to as *hkb^lacZ^*) [44]; *gsb^01155^* (referred to as *gsb^lacZ^*) [45]; and *wg^758^* (referred to as *wg^GAL4^*) provided by K. Basler. Mutants were kept over *CyO, Act-GFP,* or *TM3, Ser, Act-GFP* balancer chromosomes. *w^1118^* was often used as wild type.

### RNAi of *col*.

A 497–base pair (bp) fragment of the *col* cDNA (position 1,584–2,080 of GenBank sequence NM 080074) was amplified by PCR, sequenced, and then inserted as an inverted repeat into the pWIZ vector [46]. Transgenes, denoted *UAS-col-dsRNA,* were generated by standard methods. Six different *UAS-col-dsRNA* transgenic lines were driven by *ap^GAL4^,* and showed varying degrees of *col* wing effects (L3–L4 wing vein fusion). Two strong lines were used for CNS analysis. To enhance the *col* RNAi effect, a *UAS-Dcr-2* transgene (on the X chromosome) was used (a gift from G. Dietzl and B. Dickson).

### Immunohistochemistry.

Full-length *col* (in pET17b, provided by M. Crozatier and A. Vincent) and C-terminal part of *dimm* (amino acid residues 210–390 in pGEX-4T) were expressed in bacteria, and antibodies were raised in rabbits and guinea pigs (AgriSera, Vännäs, Sweden). Antibodies to IPNamide and proNplp1 were raised in hens against the peptide sequences NVGTLARDFQLPIPNamide and FLGRVLPPTRATASTHRSRL, respectively (AgriSera). Antibodies to proFMRFa were raised in rabbits against the C-terminal peptide sequence GAQATTTQDGSVEQDQFFGQ (Covance, Princeton, New Jersey, United States). Peptide antibodies were affinity purified, and all polyclonal sera were pre-absorbed against pools of early embryos. Other antibodies used were: guinea pig α-Deadpan (1:1,000) (provided by J. Skeath), rat monoclonal α-Gsbn (1:10) (provided by R. Holmgren), mouse monoclonal antibody (mAb) α-Col (1:250) (provided by M. Crozatier and A. Vincent), mAb α-ELAV (1:10), mAb α-Repo (1:10), mAb α-c-Myc mAb 9E10 (1:30), concentrated mAb α-β-gal mAb 40–1a (1:20), mAb α-Dac dac2–3 (1:25), mAb α-Eya 10H6 (1:250) (all from Developmental Studies Hybridoma Bank, Iowa City, Iowa, United States), rabbit α-β-gal (1:5,000; ICN-Cappel, Aurora, Ohio, United States), rabbit α-GFP (1:500; Molecular Probes, Eugene, Oregon, United States), and rabbit α-phospho-histone H3-Ser10 (pH 3) (1:2,500; Upstate/Millepore, Billerica, Massachusetts, United States). Immunolabeling was carried out as previously described [10].

### Confocal imaging and data acquisition.

Zeiss LSM 5 Confocal microscope was used to collect data for all fluorescent images (Zeiss, Oberkochen, Germany); confocal stacks were merged using LSM 5 software or Adobe Photoshop (Adobe Systems, San Jose, California, United States). Where immunolabeling was compared for levels of expression, wild-type and mutant tissue was stained and analyzed on the same slide. Bright-field images were collected on a Nikon E400 microscope with a SPOT-RT digital camera (Nikon, Tokyo, Japan). Statistical analysis was performed using Microsoft Excel (Redmond, Washington, United States). Where appropriate, images were false colored to facilitate for color-blind readers.

## Supporting Information

Figure S1 A Specific Subset of Ap Neurons, the Tvb and dAp Cells, Expresses the Nplp1 Neuropeptide Gene (1.7 MB AI).

Figure S2 *col* Is Not Regulated by Other Ap Neuron Determinants, whereas *eya* Regulates Dimm (8.2 MB AI).

Figure S3 Thoracic Neuroblast 5–6 Generates the Ap Cluster, and *col* Is Expressed in Late-Stage Thoracic NB 5–6 (8.0 MB PDF)

Text S1 Thoracic Neuroblast 5–6 Generates the Ap Cluster, and *col* Is Expressed in Late-Stage Thoracic NB 5–6 (52 KB DOC)

### Accession Numbers

The FlyBase (http://flybase.bio.indiana.edu/search) accession numbers for the genes and gene products discussed in this paper are Ap (CG8376), *col* (Flybase *knot*) (CG10197), *dac* (CG4952), *dimm* (CG8667), DopR (CG9652), *eya* (CG9554), FMRFa, (CG2346), Gbb (CG5562), *Nplp1* (CG3441), *PHM* (CG3832), *sqz* (CG5557), and Wit (CG10776).

The GenBank (http://www.ncbi.nlm.nih.gov/Genbank) accession number for the *col* cDNA from which a 497-bp fragment was taken is NM 080074.

## Abbreviations

AEL: after egg laying
CNS: central nervous system
dAp: dorsal Ap
mAb: monoclonal antibody
RNAi: RNA-interference
VNC: ventral nerve cord
