# Supplementary material for: Specification of Neuronal Identities by Feedforward Combinatorial Coding
Source: PLoS Biol. 2007 Feb 6;5(2):e37. doi: 10.1371/journal.pbio.0050037 (PMC1790951; doi:10.1371/journal.pbio.0050037)

Supplemental Figure 1

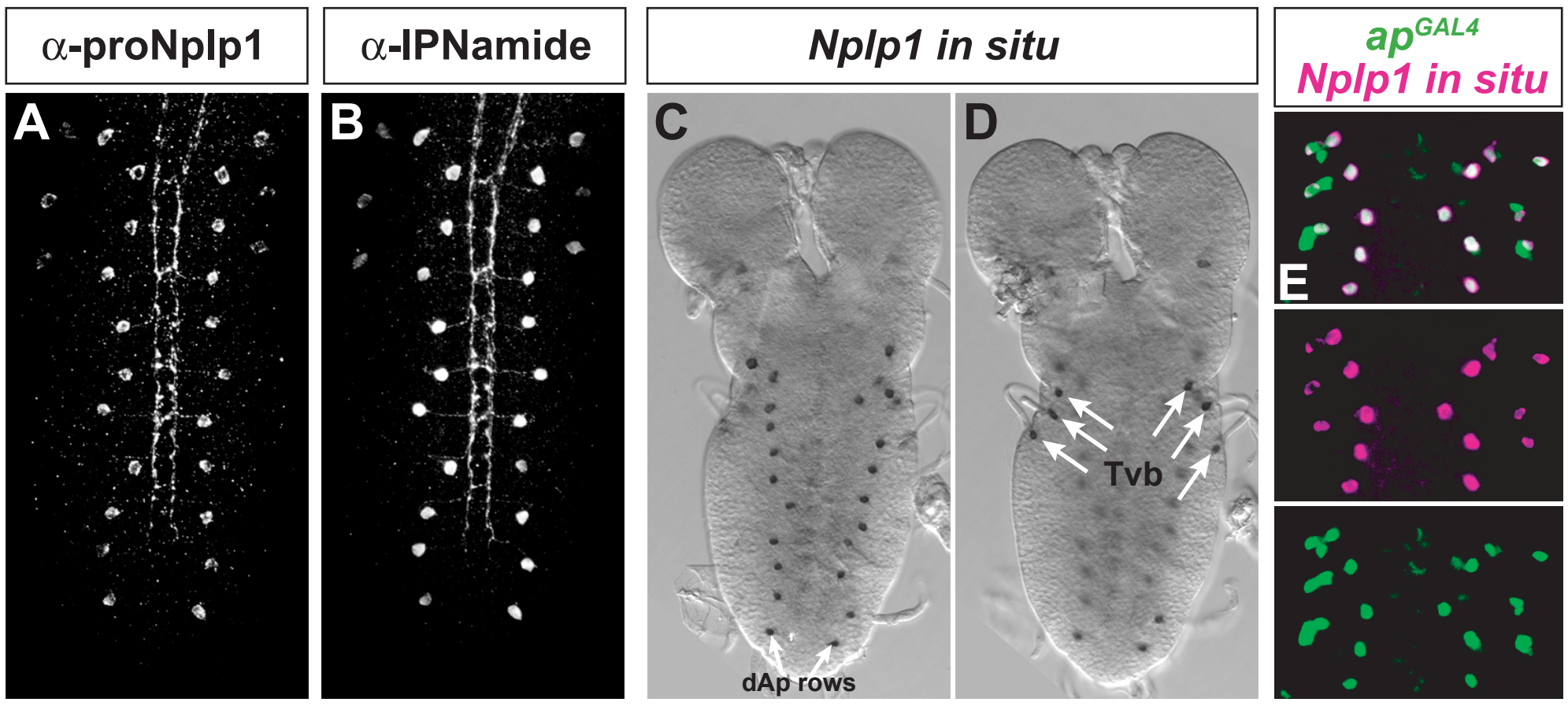

Supplement: Supplementary file 1 [file pbio.0050037.sg001.pdf]
