# Supplementary material for: Specification of Neuronal Identities by Feedforward Combinatorial Coding
Source: PLoS Biol. 2007 Feb 6;5(2):e37. doi: 10.1371/journal.pbio.0050037 (PMC1790951; doi:10.1371/journal.pbio.0050037)
Supplement: Supplementary file 4 [file pbio.0050037.sd001.doc]

**Supplemental Figure S1.** A specific subset of Ap neurons, the Tvb and dAp cells, expresses the Nplp1 neuropeptide gene. (A-D) Expression of proNplp1 (A), IPNamide (B), and *Nplp1* RNA (C, D), in a small subset of VNC cells. Overlap between *Nplp1* RNA and *ap^GAL4^* expression (E), reveals that *Nplp1* expressing cells constitute a subset of Ap neurons. All images are from 18 h AEL VNCs.

**Supplemental Figure S2.** *col* is not regulated by other Ap neuron determinants, while *eya* regulates Dimm.

(A-I) Expression of Col at 18 h AEL in wild type (A), *sqz* (B-C), *dac* (D), *wit* (E), *dimm* (F), control (G), *eya* (H) and *ap* (I). (B-C) In *sqz* mutants, Ap cluster composition is altered with typically one extra ‘Tvb’ cell in the T2/T3 segments, and several extra Ap cluster neurons and ‘Tvb’ cells in the T1 segment. As expected, this leads to extra Col expressing cells as well (arrows depict ‘Tvb’ neurons). (D, E, F) In *dac*, *wit* and *dimm* mutants, there is no effect upon Col expression (arrows depict Tvb neurons). (H) In *eya* mutants, Col expression is variably affected, and there are examples of unaffected clusters with one Col expressing cell (arrow), but frequently Col expression may either be lost or observed in two cells (see J). (I) In *ap* mutants, cluster composition is also affected and we frequently observe all 4 Ap neurons expressing Dac, including the Tvb neuron (arrow) and there is a small but significant variation of Col expression (p<0.01; n=78 hemisegments). (K-L) Expression of Dimm in *eya* and *dac* mutants reveals that *eya* but not *dac* is necessary for Dimm expression in both Tv and Tvb. (M-N) In *dimm* mutants, there is no effect upon Eya or Dac expression. FMRFa and Nplp1 is reduced but still detectable. (J, O) Quantification of the number of Col and Dimm expressing cells per Ap cluster, in control and mutant backgrounds (n>31 hemisegments). (J) In *eya* mutants, Col expression is not significantly altered, but the large standard deviation underscores the variability in Col expression. Asterisks denote significant alteration compared to control (students t-test, p<0.01). All images are from 18 h AEL VNCs.

Genotypes: (A) *w^1118^*. (B-C) *sqz^DF^/sqz^ie^*. (D) *dac^3^*/*dac^4^*. (E) *wit^A12^*/*wit^B11^*. (F, M, N) *dimm^p1^*/*dimm^rev4^*. (L) *dac^3^*/*dac^4^*, *ap^GAL4^*; *UAS- mEGFP^F^/+*. (G) *ap^GAL4^*/+; *UAS-mEGFP^F^/+*. (H, K) *eya^Cli-IID^, ap^GAL4^*/*eya^10^*; *UAS-mEGFP^F^/+.* (I) *ap^p44^*/*ap^GAL4^*; *UAS-nmEGFP/+*. (J, O) control is *ap^GAL4^*/+; *UAS-mEGFP^F^/+*.

**Mapping of the Ap cluster to neuroblast 5-6.**

We predicted that the Ap cluster would be generated from NB 5-6 or NB 6-4, and as expected, we found overlap between Col, Eya and *ap* expression and both *gsb^lacZ^* or Gsbn, markers for rows 5 and 6 [1-4] (not shown). This overlap was observed in cells in the anterior-most region of *gsb^lacZ^* and Gsbn expression, suggesting a row 5 origin for the Ap cluster. This was verified by overlap between Col, Eya, *ap*, and *wg^GAL4^*, a specific marker for row 5 (Figure S3D) [5]. The lateral-most position of Col/Eya cells within row 5 supported the idea that the lateral-most row 5 neuroblast, NB 5-6, indeed generates the Ap cluster. This notion was confirmed by the non-overlap between Col and *huckebein* (*hkb^lacZ^*) expression, a marker for NB 5-5 and NB 5-4 (Figure S3G) [6,7], and by the overlap between Col and *ladybird-lacZ* (*K-lacZ*), a specific marker for NB 5-6 (Figure S3H) [8]. To map the expression of Col within the NB 5-6 lineage itself, we used phospho-Histone H3-Ser10 (pH3) as a marker for mitotic cells [9], and could typically find one pH3/Col expressing cell at stage 14 (Figure S3C). At stage 14 and stage 13, we observe overlap between Col and Deadpan, a neuroblast/GMC marker (Figure S3B) [10]. At stage 12 we find no Col expression in any row 5 cells (Figure S3A). Previous studies have revealed that NB 5-6 is generated in the first wave of neuroblasts, delaminating at late stage 8 [11]. It rapidly begins to generate a mixed lineage of, in the thorax, 10-14 neurons and 3-5 glia [12,13]. Given the rapid pace of these events, and given the relatively late appearance of Ap cluster neurons, we anticipated finding both neurons and glia in the lateral-most area of row 5 prior to the appearance of Ap cluster neurons. This was confirmed by the finding of lateral Elav/*wg^GAL4^* and Repo/*wg^GAL4^* expressing cells, markers of neurons and glia, respectively, at stage 13 (Figure S3I, S3J).

In summary, Col expression commences within NB 5-6 at stage 13, remains on in the neuroblast at stage 14, but also appears in a GMC at this stage, as evident from the simultaneous observation both of smaller and more dorsally located Col/pH3 expressing cells, and large ventrally located Col/Deadpan expressing cells at this stage. At late stage 14, the first two *ap* cluster neurons are born, as evident from Col expressing, non-pH3 expressing, smaller cells in a more dorsal focal plane. Col expression in these two Ap cluster neurons is often observed simultaneously with a more ventral pH3/Col cell, presumably a second Col expressing GMC. At early stage 15, there are no Col/pH3 cells visible and instead two more Ap cluster neurons are added, as evident from the appearance of four Col, Dac, Eya, ap, sqz, expressing cells in a dorsal focal plane.

**Supplemental Figure S3.** Thoracic neuroblast 5-6 generates the Ap cluster, and *col* is expressed in late stage thoracic NB 5-6.

(A-J) Thoracic segments, and Ap clusters (K, L) stained for the markers listed at bottom. Dashed lines depict the VNC midline. (A) At stage 12, there is no apparent expression of Col in row 5. (B) At stage 13, Col expression is evident in the lateral-most row 5 neuroblasts, evident from the overlap with Deadpan (Dpn; a neuroblast/GMC marker) and the anterior domain of Gsbn expressing cells (Gsbn marks rows 5 and 6). (C) At stage 14, expression of Col overlaps with the mitotic marker phospo-Histone3 (pH3) in lateral-most row 5 cells. (D-E) At stage 15, Col expression is evident in all 4 Ap cluster neurons, evident from overlap with Eya and *ap^lacZ^*. The Ap cluster is generated from the later-most region of row 5, evident from overlap with *wg^GAL4^* (a marker for row 5). (K-L) At stage 16 and 17, expression of Col is rapidly downregulated and only apparent in the Tvb neuron, evident from non-overlap with Dac. (G) At stage 14, expression of Col does not overlap with that of *hkb^lacZ^*, a marker for more medial row 5 neuroblasts i.e. NB 5-5 and NB 5-4. (H) Col expression does overlap with expression of *ladybirdb-lacZ* (*K-lacZ*), a marker for NB 5-6. (I-J) At stage 14, there are already neurons and glia present in the lateral-most region of row 5, as evident by overlap between *wg^GAL4^* and Elav (I), a marker for neurons, and Repo (J) a marker for glia. Propidium iodide (PI) was used to reveal all cell nuclei.

1. Gutjahr T, Patel NH, Li X, Goodman CS, Noll M (1993) Analysis of the gooseberry locus in Drosophila embryos: gooseberry determines the cuticular pattern and activates gooseberry neuro. Development 118: 21-31.

2. Buenzow DE, Holmgren R (1995) Expression of the Drosophila gooseberry locus defines a subset of neuroblast lineages in the central nervous system. Dev Biol 170: 338-349.

3. Duman-Scheel M, Li X, Orlov I, Noll M, Patel NH (1997) Genetic separation of the neural and cuticular patterning functions of gooseberry. Development 124: 2855-2865.

4. Skeath JB, Zhang Y, Holmgren R, Carroll SB, Doe CQ (1995) Specification of neuroblast identity in the Drosophila embryonic central nervous system by gooseberry-distal. Nature 376: 427-430.

5. Chu-LaGraff Q, Doe CQ (1993) Neuroblast specification and formation regulated by wingless in the Drosophila CNS. Science 261: 1594-1597.

6. Chu-LaGraff Q, Schmid A, Leidel J, Bronner G, Jackle H, et al. (1995) huckebein specifies aspects of CNS precursor identity required for motoneuron axon pathfinding. Neuron 15: 1041-1051.

7. Bossing T, Technau GM, Doe CQ (1996) huckebein is required for glial development and axon pathfinding in the neuroblast 1-1 and neuroblast 2-2 lineages in the Drosophila central nervous system. Mech Dev 55: 53-64.

8. De Graeve F, Jagla T, Daponte JP, Rickert C, Dastugue B, et al. (2004) The ladybird homeobox genes are essential for the specification of a subpopulation of neural cells. Dev Biol 270: 122-134.

9. Adams RR, Maiato H, Earnshaw WC, Carmena M (2001) Essential roles of Drosophila inner centromere protein (INCENP) and aurora B in histone H3 phosphorylation, metaphase chromosome alignment, kinetochore disjunction, and chromosome segregation. J Cell Biol 153: 865-880.

10. Bier E, Vaessin H, Younger-Shepherd S, Jan LY, Jan YN (1992) deadpan, an essential pan-neural gene in Drosophila, encodes a helix-loop-helix protein similar to the hairy gene product. Genes Dev 6: 2137-2151.

11. Doe CQ, Technau GM (1993) Identification and cell lineage of individual neural precursors in the Drosophila CNS. Trends Neurosci 16: 510-514.

12. Schmidt H, Rickert C, Bossing T, Vef O, Urban J, et al. (1997) The embryonic central nervous system lineages of Drosophila melanogaster. II. Neuroblast lineages derived from the dorsal part of the neuroectoderm. Dev Biol 189: 186-204.

13. Schmid A, Chiba A, Doe CQ (1999) Clonal analysis of Drosophila embryonic neuroblasts: neural cell types, axon projections and muscle targets. Development 126: 4653-4689.
